# The Formation of Protein–Chitosan Complexes: Their Interaction, Applications, and Challenges

**DOI:** 10.3390/foods13223572

**Published:** 2024-11-08

**Authors:** Yufeng Xie, Jiaqi Ding, Yue Li, Pengfei Wei, Shiying Liu, Rui Yang

**Affiliations:** 1 College of Food Science and Engineering, Harbin University, Harbin 150086, China; 2 Liquor Making Biological Technology and Application of Key Laboratory of Sichuan Province, Yibin 644005, China; 3 College of Food Science and Engineering, Tianjin University of Science and Technology, Tianjin 300457, China

**Keywords:** chitosan, protein, functional properties, applications

## Abstract

Protein–polysaccharide interactions have been a subject of considerable interest in the field of food science. Chitosan is the most prominent and naturally occurring polysaccharide with a positive charge, and its hydroxyl and amino groups facilitate protein–chitosan interactions due to their diverse biochemical activities. The complexation of chitosan enables the modification of proteins, thereby enhancing their value for applications in the food and nutrition industry. This paper presents a summary of the complexes formed by chitosan and different proteins, such as lactoglobulin, egg white protein, soybean isolate protein, whey isolate protein, and myofibrillar protein, and systematically describes the modes of interaction between proteins and chitosan. The effects of protein–chitosan interactions on functional properties such as solubility, emulsification, antioxidant activity, and stability are outlined, and the potential applications of protein–chitosan complexes are discussed. In addition, the current challenges associated with the formation of protein–chitosan complexes and potential solutions to these challenges are highlighted. This paper provides an overview of the current research progress on the interaction of proteins with chitosan and its derivatives in the food industry.

## 1. Introduction

Protein is an essential biomolecule present in a multitude of foods and exhibits a diverse array of properties. Proteins have amphiphilic, emulsifying, foaming, film-forming, and water retention properties, and they are widely used in food stability and emulsification. The quality of food largely depends on the use of proteins as stabilizers and emulsifiers [1,2]. Polysaccharides also play an important role in the functional properties of foods by interacting with various food components. The interactions between food protein and polysaccharides are particularly prevalent. This is because electrostatic interactions, covalent bonds, and some non-covalent bonds are highly likely to occur between polysaccharides and proteins [3]. Protein–polysaccharide interactions can be used not only to assess changes in proteins during processing, transport, and storage but also to modify proteins to improve their functional properties.

**Figure 1:**
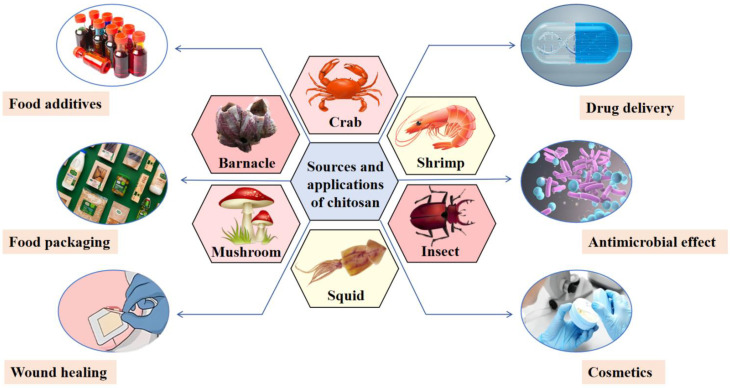
Sources and applications of chitosan. Chitosan is mainly derived from the shells of shrimps, crabs, and insects and is used in cosmetics, food, and other applications.

Chitosan is primarily derived from insects and crustaceans, as shown in Figure 1, and is the second largest natural polysaccharide in nature after cellulose. It is a polymer derived from the deacetylation of chitin and is soluble at acidic pH [4]. Chitosan is antimicrobial, biocompatible, biodegradable, and biorejective [5]. The aforementioned properties of chitosan have led to its frequent use in conjunction with proteins, with the aim of increasing the solubility and stability of said proteins. This is achieved through the formation of complexes used in the production of emulsions, gels, and other food ingredients. Figure 1 shows an illustrative example of this process. Chitosan has been used in a variety of fields, including the food industry, agriculture, and biomedicine [6]. The simultaneous presence of chitosan and protein results in the formation of complexes that are more effective than those observed when the two components are employed in isolation. These complexes have the potential to enhance the quality and functional properties of food products [7]. For instance, biopolymers formed by chitosan complexed with myofibrillar protein, phycocyanin, and egg white protein exhibit enhanced emulsification and stability properties [8,9,10]. Furthermore, chitosan can facilitate the solubility of protein following interaction with zein alcohol-soluble protein and β-lactoglobulin [11,12]. Moreover, protein–chitosan complexes are widely used in food applications, such as whey isolate protein–chitosan complexes used to develop antimicrobial aerogels for chicken preservation, and cod protein–chitosan complexes can be used to construct food-grade emulsion delivery systems for astaxanthin delivery [13,14]. At present, protein–chitosan complexes have attracted extensive attention due to their excellent functional properties and wide range of applications.

This paper reviews the mode of interaction between protein and chitosan and its effect on the functional properties of protein–chitosan complexes. These properties mainly include solubility, emulsification, stability, gelation, and antioxidant activity. The interaction mode between protein and chitosan is mainly categorized into non-covalent and covalent interactions. Furthermore, the paper describes the extensive uses of protein–chitosan complexes in microencapsulation, composite films and coatings, antimicrobial aerogels, food-grade emulsion delivery systems, and other applications in the food industry. This paper also highlights the challenges faced by protein–chitosan complexes in the production of food products. In particular, issues such as stability, safety, compatibility, and sustainability need to be further explored in relation to protein–chitosan complexes.

## 2. Mode of Interaction Between Protein and Chitosan

**Figure 2:**
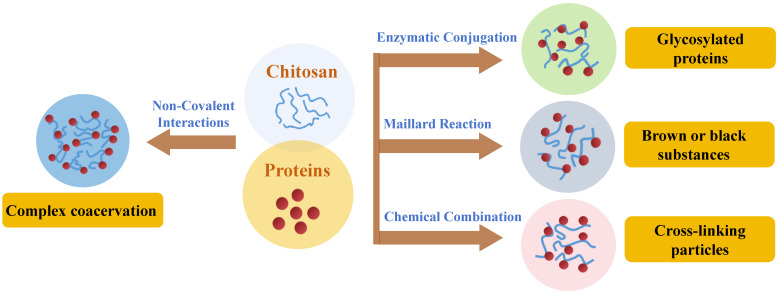
The interactions between protein and chitosan are basically classified as non-covalent polymerization, chemical cross-linking, enzymatic glycosylation, and Maillard reaction.

The modes of interaction for the formation of protein–chitosan systems include non-covalent and covalent interactions. Non-covalent interactions, which include electrostatic interactions, hydrophobic interactions, hydrogen bonding, and van der Waals forces, are formed spontaneously when substances are mixed, and they can contribute to the formation of emulsion stabilizers, gels, thin films, and edible protective coatings. Most non-covalent interactions are weak and reversible and can be modified by adjusting factors such as pH, ionic strength, and temperature [15,16]. Covalent interactions are processes that create covalent bonds through Maillard reactions, enzyme-catalyzed reactions, and chemical cross-linking reactions that make protein–chitosan complexes more stable [17]. However, in order to achieve the desired reaction, it is necessary to adjust the reaction conditions, such as pH, temperature, ionic strength, and reaction time [18]. The primary binding modes of protein and chitosan are illustrated in Figure 2. These modes are broadly classified into physical copolymerization, chemical cross-linking, enzymatic glycosylation, and the Maillard reaction. The following sections provide a detailed analysis of the interaction between protein and chitosan.

### 2.1. Non-Covalent Interactions

The non-covalent interactions between protein and chitosan are primarily designed to exploit the charged properties of the two polyelectrolytes. Electrostatically attractive interactions are the primary driving force. Hydrophobicity, hydrogen bonding, and spatial interactions are also involved [19,20]. Proteins are amphipathic, meaning that their surface charge depends on the pH of the solution. Although the net charge of a protein is zero at its isoelectric point, its surface still has positive and negative regions. This suggests that it may be involved in electrostatic interactions of attraction or repulsion [21]. The charge of chitosan is determined by the amino side groups, with a pKa of approximately 6.5. At relatively low pH values, chitosan can be dissolved in water after protonation of the amino group, which imparts a positive charge. However, with the increase in pH, more chitosan is required to wrap around the protein due to the decrease in the charge of chitosan and electrostatic repulsion, and the complex is prone to sedimentation at a high pH [22,23]. The electrical properties of protein and chitosan can be exploited to design a range of complexes that meet the specific requirements of the food industry.

The non-covalent interactions between protein and chitosan are mainly based on the charged nature of both. By adjusting pH and ionic strength, soluble complexes or cohesive layers can be formed for use in foods. The pH-induced interaction between gliadin and chitosan was investigated. At pH 5.0, the gliadin–chitosan soluble complex presented spherical nanoparticles with a particle size of 570.42 nm, polymer dispersity index (PDI) of 0.191, and ζ potential of +19.2 mV, showing good uniformity and dispersity. In addition, the encapsulation rate was as high as 85.11%, and the release rate of curcumin during pepsin and trypsin digestion was reduced [24]. The complexes of soybean isolate protein (SPI) with chitosan (CS) were subjected to heating, and it was observed that the heated SPI-CS complexes exhibited enhanced acidic solubility [25]. The most likely explanation for this phenomenon is that more negatively charged carboxyl groups on the proteins were exposed during the heating process, and these charged carboxyl groups interacted with the amino groups of chitosan, resulting in an increase in the isoelectric point from 4.4 ± 0.1 (SPI) to 5.5 ± 0.1 (SPI-CS) and a significant increase in the solubility at pH 4.0 [25]. The same conclusion was reached that soybean globulin–chitosan mixtures exhibited a significant increase in potential positivity and stability upon heating at pH 3.3 [26]. Some therapeutic proteins become more stable after binding with chitosan through electrostatic interaction and can be free from the influence of chemical and physical factors so as to improve bioavailability. In addition, the non-covalent interaction between protein and chitosan can improve the stability of protein in acidic and thermal environments [27]. It was demonstrated that the whey protein–chitosan composite system has excellent antioxidant and emulsifying properties, and it is beneficial for the development of this composite system in the field of food additives, food packaging, and drug delivery [28].

### 2.2. Covalent Interactions

Non-covalent bonds are typically reversible (physical) interactions, while covalent bonds are irreversible (chemical) interactions. In certain applications, it is more necessary to form covalent bonds because they produce stronger and more durable interactions [29]. Covalent interactions between protein and chitosan employ either a Maillard reaction or an enzyme-catalyzed reaction (such as transglutaminase or tyrosinase). The resulting conjugates retain the advantages of both substrates in a single entity. The following sections describe these methods in detail.

#### 2.2.1. Maillard Reaction

The Maillard reaction is a non-enzymatic browning, a complex reaction between carbonyl and amino compounds, and ultimately produces a brown or black melanin-like substance [30,31]. The Maillard reaction between protein and chitosan occurs mainly between the amino groups of proteins (particularly the ε-amino groups of lysine residues) and the carbonyl groups of the N-acetylglucosamine moiety of chitosan [32]. There are many factors affecting the Maillard reaction, including the type of polysaccharides and protein, the molecular weight of the polysaccharides, and the ratio of protein to polysaccharides [33,34]. The effect of molecular weight on the functional properties of β-lactoglobulin Maillard products was investigated [34]. The activity of the Maillard reaction was related to the molecular weight of the sugar. The conjugates formed with enzymatically depolymerized chitosan (1.3 kDa) showed the sharp formation of the end product. In general, the lower the molecular weight, the higher the activity of the Maillard reaction. However, the high reaction activity leads to lower nutritional value. Conversely, the molecular weight of sugar is positively correlated with the chain length of sugar. The longer the chain length, the greater the spatial resistance, which slows down the process and prevents the production of harmful substances [35].

#### 2.2.2. Enzymatic Glycosylation

Another type of covalent interaction is the enzyme-catalyzed binding of proteins to chitosan, also known as glycosylation. Protein glycosylation is the covalent attachment of proteins to monosaccharides or glycans. It is one of the most common methods of protein modification, and it can significantly alter the structure, properties, and function of proteins. The most commonly used enzymes are transglutaminase and tyrosinase. They can catalyze the reaction of active groups between protein and chitosan to form covalent bonds. Enzymatic glycosylation has a number of advantages over chemical modification of the protein, including a shorter reaction time, greater control over the degree of glycosylation, the ability to attach sugars to specific sites on the protein, and the ability to eliminate toxic reagents [36]. It has been demonstrated that these enzymes can significantly enhance the functional properties of protein. The following paragraphs present two microbial enzymes that are safe and widely used in the food industry.

##### Transglutaminase

Transglutaminase (TGase) is an enzyme that modifies proteins by amine incorporation, cross-linking, and deamidation. The use of transglutaminase to catalyze protein glycosylation has been reported since the early 1980s [37,38]. At that time, guinea pig liver transglutaminase, which is Ca^2+^-dependent, was used. The currently used microbial transglutaminase (MTGase) does not rely on Ca^2+^, which makes it safer and less expensive. It can also be produced in large quantities. In addition, microbial transglutaminase was employed to glycosylate zein with chitosan (MW 1500 Da), and 97.48 mg of glucosamine was covalently conjugated to 1 g of zein. This process significantly increased the solubility and antioxidant activity of zein in vitro while concomitantly reducing its surface hydrophobicity [39]. It is crucial to recognize that intramolecular and intermolecular cross-linking of the protein occurs simultaneously in cases where the protein contains a high number of lysine residues. For proteins with a high lysine content, such as soybean 11s globulin, pea 11s globulin, casein, etc., to prevent intramolecular cross-linking from affecting protein glycosylation, the protein can be alkylated first [36]. Corn gluten is a protein with low lysine content. Its enzymatic glycation reaction mainly occurs with sugars that have reactive primary amino groups, and the probability of internal occurrence is low. Therefore, corn protein may be a suitable substrate for enzymatic glycation to prepare glycoproteins [39]. The use of transglutaminase to couple chitosan to zein significantly enhances the water solubility and emulsification of zein [40]. Moreover, glycosylated caseinate has enhanced surface hydrophobicity, water-binding capacity, rheological properties, and in vitro digestibility compared to the original caseinate [41].

##### Tyrosinase

Tyrosinase is an enzyme that contains copper and is commonly found in nature. It catalyzes the conversion of phenolics to o-quinones, thereby causing the ripening or discoloring of the skins of fruits and vegetables. Tyrosinase can also be used to link protein to the biopolymer chitosan, preserving the biological activity of the protein [42]. The reaction mechanism of tyrosinase to protein-bound tyrosine residues involves hydroxylation to 3,4-dihydroxyphenylalanine, followed by oxidation to the corresponding o-quinone [43]. These o-quinones are active compounds and can be condensed with each other or react with nucleophiles. Protein can bind to amines and sulfhydryl groups of amino acid residues or amine groups of chitosan [44]. Enzymatic protein–polysaccharide grafting has great potential for the production of a new range of bio-based, environmentally friendly polymers. Tyrosinase is used in the preparation of spike protein conjugates due to the high reactivity of dopaquinone with the amino groups present in chitosan [45]. The peptides are enzymatically immobilized on gold nanoparticles. The amino groups of chitosan are linked to DOPA–quinone, and the DOPA–quinone is produced from tyrosine by tyrosinase. The study demonstrated that the tyrosinase-mediated linkage between peptides and chitosan-coated particles played a crucial role in the production of functionalized nanoparticles [46].

Although both transglutaminase and tyrosinase are enzyme preparations commonly used for glycosylation, their mechanisms of action are different: TGase catalyzes the formation of a peptide bond between the γ-carboxamide of a glutamine residue and the ε-amino groups of lysine residues, whereas tyrosinase catalyzes the reaction of oxidized substances with chitosan amino groups to form imide linkages or secondary amines [47]. They have been used to catalyze the cross-linking of gelatin and chitosan, respectively, to form gels. The gel formed by transglutaminase showed better mechanical properties [18].

### 2.3. Crosslinking Agent

Chemical crosslinking agents can be divided into five main categories: aldehyde crosslinking agents, sodium tripolyphosphate crosslinking agents, vanillin crosslinking agents, propylene oxide crosslinking agents, and methylene bisacrylamide. Each of these agents has distinct properties. For example, glutaraldehyde is a commonly used substance, but it is toxic and unsuitable for fruit preservation. In contrast, tripolyphosphate is an ionic crosslinker that can only be used when chitosan is protonated. Vanillin is derived from plants, but its cross-linking bonds are easily broken. As a result, the use of the aforementioned chitosan-based crosslinkers is limited.

Genipin can react with the amino group of the enzymes, leading to an increase in cross-linking groups. It can be cross-linked with chitosan as an immobilization carrier for β-D-galactosidase from Aspergillus oryzae, and the resulting particles were more thermally stable, acid-resistant, and mechanically resistant [48]. Genipine can be used as a replacement for conventional reagents like glutaraldehyde in enzyme immobilization. Compared to glutaraldehyde, genipin is much less cytotoxic [49]. Moreover, genipin crosslinked chitosan microspheres have been investigated to immobilize β-galactosidase from *Aspergillus oryzae*. The resulting immobilized enzyme has high stability, tolerance, and suitability for continuous production [50]. The effects of three different cross-linking agents (tripolyphosphate, phytate, and sodium phytate) on the properties of gliadin–chitosan composite nanoparticles were also investigated [51]. The presence of chitosan was able to increase the encapsulation rate of curcumin in nanoparticles, but the cross-linking agents were able to enhance the hydrogen bonding and electrostatic interactions of the nanoparticles. Specifically, the tripolyphosphate-crosslinked nanoparticles had the highest encapsulation rate of 86.1%. Compared with curcumin–gliadin–chitosan nanoparticles, the encapsulation efficiency was increased by 23.5%. Phytic acid and phytate-crosslinked nanoparticles had better thermal and UV stability, while sodium phytate had a better potential to protect curcumin in vitro [51]. Therefore, the choice for these three cross-linking agents should be made according to the purpose of preparing specific composite nanoparticles.

## 3. Effect of Interaction Between Protein and Chitosan on Functional Properties

Protein and chitosan are widely used in food applications and pharmaceutical and bioengineering industries due to their multifunctional properties. In various food systems, protein and chitosan play a pivotal role in determining product stability, antioxidant properties, viscosity, texture, and flavor. However, the functional properties of protein–chitosan complexes are distinct from those of single macromolecular substances, and their complexes exhibit superior functional properties.

### 3.1. Solubility

The limited solubility of proteins can have a significant impact on their functional properties, limiting their use in the food industry. Currently, various methods, such as protease hydrolysis, deamidation, and glycosylation, have been developed to increase the solubility of proteins [52]. Among these approaches, glycosylation stands out as the most widely used technique due to its effectiveness on a wide range of proteins. For example, it has been shown that zein hydrolysate, after glycosylation with chitosan, exhibits a significant increase in solubility within the pH range of 5–7 [11]. The study investigated the effect of interaction between β-lactoglobulin and chitosan on solubility in aqueous solutions. The results showed that chitosan can form soluble complexes with β-lactoglobulin at pH 4.0, which increases the solubility of the protein. This phenomenon can be attributed to the electrostatic forces that arise between biopolymers of opposite charges when subjected to changes in pH [12].

### 3.2. Emulsification Degree

As amphiphilic molecules, proteins are able to adsorb at the oil–water interface to stabilize emulsions, but their stability is easily affected by temperature, pH, ionic strength, and other factors. However, when combined with polysaccharides, the solubility and stability of proteins are increased, which can improve the emulsification properties of proteins [53]. Therefore, protein–polysaccharide complexes with strong emulsifying properties have become a hot research topic nowadays. A previous study has found that potato isolate proteins modified and complexed with chitosan resulted in complex-stabilized emulsions with better emulsification properties [54]. The latest study investigated the emulsification properties of myofibrillar protein–chitosan complexes under acidic conditions (pH 3–6) and showed that chitosan enhanced the emulsification properties of myofibrillar protein [55]. In addition, a new type of egg white protein chitosan bilayer emulsion was obtained by ultrasound and glutamine aminotransferase modification technology [56]. When the double emulsion contained chitosan (0.6%, *v*/*v*), the zeta potential of the double emulsion was −1.1 mV with a small particle size (56.87 µm), and the delamination index was 16.3%. This uniform droplet dispersion is suitable for the transportation of food-grade bioactive substances (such as β-carotene) [56].

### 3.3. Gel

With the rapid development of the modern food industry and the general improvement of people’s understanding of healthy diets, people gradually prefer gel food because it has many advantages over other types of food. These include a high water content, low energy, and an attractive texture. Different types of complexes tend to have more advantages in the preparation of binary food gels than the same types of complexes (such as polysaccharide–polysaccharide complexes and protein–protein complexes) [57]. It is commonly believed that protein–chitosan gels are formed when proteins are denatured during heating and subsequently aggregated and crosslinked with polysaccharides to form a gel. The main reason is the interaction between protein and chitosan [58]. The formation of the composite gel layer of pea protein isolate–chitosan (FPPI/CH) was primarily determined by electrostatic and hydrophobic interactions [59]. Hydrogen bonding also played a role in the occurrence of electrostatic complexation, resulting in the formation of the cohesive layer of FPPI/CH complexes. Specifically, the FPPI/CH formed by high molecular weight chitosan (HMW) (310–375 kDa, >75% deacetylated degree) and the pea protein isolate showed a uniform microstructure [59]. Pre-aggregates of soy isolate protein–naringenin complexes were prepared by ohmic heating with the addition of chitosan to form protein gels. The addition of chitosan increases the cross-linking point and the three-dimensional network structure of the gel, improving the quality and performance of the gel [60].

### 3.4. Rheological Properties

**Figure 3:**
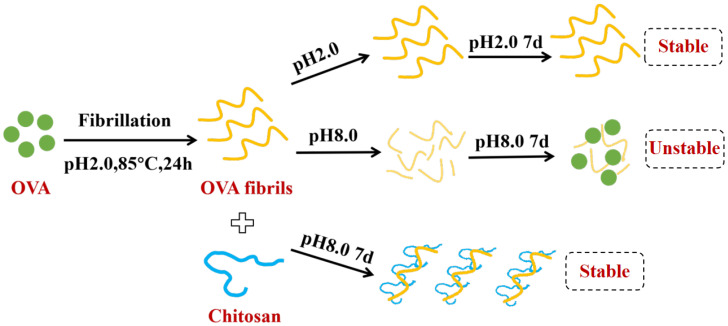
Effect of chitosan on the rheological properties of ovalbumin (OVA) under storage at pH above the isoelectric point [63].

Rheological properties refer to the deformation and flow properties of substances under external forces. A previous study revealed that the viscosity of the myofibrillar protein–chitosan complex was higher than that of myofibrillar protein and chitosan alone; as the mixture ratio of protein to chitosan decreased from 10:1 to 1:1, the viscosity of the complex increased [61]. Furthermore, non-covalent interactions between the complexes, such as hydrogen bonds, were identified as another factor that increases viscosity [62]. Because the acetyl group is closely related to the hydrophobicity of polysaccharides, the degree of deacetylation (DD) significantly affects the biological activity of chitosan, and the viscosity of chitosan mixture with high DD is higher. Therefore, the creep recovery test showed that the condensed layer formed by high DD chitosan and whey protein isolate had a denser and stronger structure. This is because the higher the DD value, the more hydrogen bonds formed in the composite, and the higher the viscoelastic modulus. It was demonstrated that the viscosity of ovalbumin (OVA) fibrils decreased significantly following storage at pH 8.0 for 7 days. However, the viscosity of OVA cellulose gradually increased with the addition of chitosan, as shown in Figure 3. The study also revealed that the G′ value of OVA fibrils decreased when stored under alkaline conditions for seven days. However, the storage modulus of OVA fibrils exhibited a gradual increase with the addition of chitosan [63]. Carboxymethylchitosan (CMC) is a water-soluble derivative of chitosan with the same biocompatibility and biodegradability as chitosan. The addition of CMC to the soy protein isolate Pickering emulsion resulted in higher G′ and G″ values. In addition, the viscosity of the emulsion increased as a consequence of the CMC addition, leading to improved emulsion stability [64].

### 3.5. Antioxidant Activity

Oxidation reactions usually occur during food processing and storage. Lipid oxidation leads to bad flavor and color changes, while protein oxidation changes the texture, digestibility, and functional properties of food [65]. Specifically, the Maillard reaction-involved browning products have been proven to exhibit antioxidant activity, and the antioxidant activity can be affected by pH and temperature [66]. When chitosan and fibrils were combined with curcumin, these complexes showed significantly improved antioxidant activity (DPPH free radical scavenging activity and reducing power) compared to curcumin alone (*p* < 0.05). This may be due to the combination of chitosan and fibrils with curcumin to form a bi-continuous polymer through electrostatic interactions, which increases the repulsive force between the fibrils, resulting in greater stability of the delivery system [67]. Algae oil is rich in polyunsaturated fatty acids, which are important for the human body, but it is also sensitive to oxygen and temperature. A previous study investigated the use of soybean protein and chitosan to create antioxidant microcapsules for delivering algal oil [68]. The optimal chitosan/soybean protein complexation pH was 6.0, and the optimum complexation ratio was 0.125 (g/g). This resulted in a significant improvement in the oxidative stability of the oil. The enhanced stability is attributed to the antioxidant properties of chitosan and the oxygen barrier provided by the composite coagulation, which increases protection against oxidation [68].

### 3.6. Reduce Protein Allergen

Food allergy has become a serious health problem globally. It is an IgE-mediated allergic reaction to specific components of food, which can be life-threatening in allergenicity. Protein allergenicity is determined by the amino acid sequence and conformation of the protein. The Maillard reaction occurs when the carbonyl group in the reducing sugar interacts with the ε-amino group of the lysine residue in the protein, leading to a series of complex chemical reactions. These reactions can result in the loss of lysine residues and alterations to the protein structure, which may contribute to allergenicity. Further hydrolysis of chitosan results in chitosan oligosaccharides (COS), which have better solubility than other polysaccharides. Tropomyosin (TM) is rich in lysine and has a high reactivity to the Maillard reaction, thus affecting its allergenicity. The Maillard reaction of chitosan, ribose, and galactose oligosaccharide with tropomyosin was carried out to remove TM from shrimp [69]. It was found that COS had the best effect on eliminating TM allergens within 4 h, while ribose and galactose showed an obvious effect after 8 h, which may be attributed to the fact that chitosan significantly alters the peptide structure by altering the β-folding [69]. A previous study coupled bovine β-lactoglobulin (βLG) with oligosaccharides via the Maillard reaction, and it was found that there was no significant change in the conformation of βLG, but the enzyme-linked immunosorbent assay demonstrated that the binding of oligosaccharides resulted in an effective decrease in the allergenicity of βLG [70]. The above studies suggest that chitosan and its derivative, such as COS, have great prospects in reducing protein allergenicity.

### 3.7. Stability

**Figure 4:**
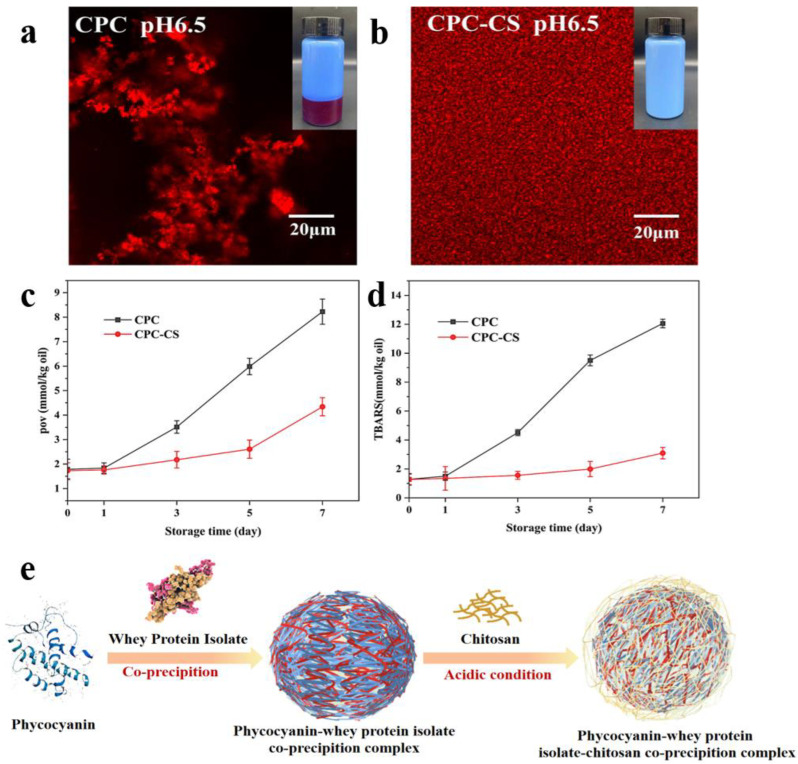
(**a**) Laser confocal scanning micrographs and photographs of a phycocyanin-stabilized emulsion at pH 6.5. (**b**) Laser confocal scanning micrographs and photographs of phycocyanin–chitosan complex-stabilized emulsion at pH 6.5. (**c**) Peroxide value of phycocyanin and phycocyanin–chitosan emulsion. (**d**) Thiobarbituric acid of phycocyanin and phycocyanin–chitosan emulsions [9]. (**e**) Mechanism diagram of the formation of whey protein isolate–phycocyanin–chitosan complex [71].

Phycocyanin is a natural pigment protein with a variety of physiological functions, but its poor emulsification, easy degradation under acidic conditions, and instability at high temperatures limit food applications. The novel phycocyanin–chitosan complexes were developed to improve the stability of the algal blue protein [9]. The laser confocal scanning micrographs and photographs of the emulsions stabilized by observation of phycocyanin and phycocyanin–chitosan complexes are shown in Figure 4a,b. At pH 6.5, the complex-stabilized emulsions contained more uniformly distributed oil droplets, indicating that the addition of chitosan significantly improved the stability of the emulsions and inhibited the aggregation of oil droplets. Furthermore, the oil droplets in the phycocyanin emulsions aggregated and were exposed to air after prolonged storage, making them highly susceptible to oxidation. The oxidation rate of the stabilized emulsion of phycocyanin–chitosan complexes was significantly reduced after the addition of chitosan, as illustrated in Figure 4c,d. An innovative method is to combine algal blue protein with whey protein, separated by protein co-precipitation, and then coat the resulting mixture with chitosan to form composite particles with colloidal stability [71]. Figure 4e illustrates the mechanistic process of the formation of this composite material. This method enabled the algal blue protein to maintain its color stability under both acidic and heating conditions. This may be attributed to the chitosan coating mitigating the electrostatic repulsion and minimizing the protein structural alterations induced by transient heating, thereby protecting the chromophore. Mixed-layer emulsions were prepared using myofibrillar fibrin–chitosan electrostatic complexes to protect and deliver astaxanthin. The mixed-layer emulsions prepared with fibronectin–chitosan complexes exhibited greater stability at pH 3, 5, and 7 and temperature changes (30, 50, and 80 °C), with a more uniform distribution. In addition, a higher astaxanthin retention (69.11%) was obtained in mixed-layer emulsions after exposure to UV-light irradiation for 8 h [10].

## 4. Application of Protein–Chitosan Complexes

**Figure 5:**
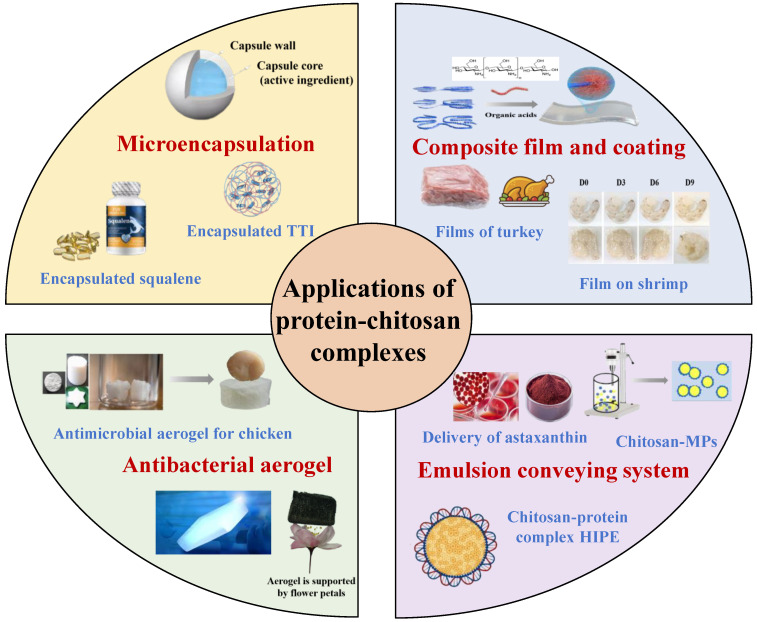
Applications of protein–chitosan complexes in the food sector, including microencapsulation, composite films and coatings, antimicrobial aerogels, and food-grade emulsion delivery systems.

Protein and chitosan have high nutritional value and functional properties, and the interaction between them has a significant impact on the food industry. The interaction between protein and chitosan can enhance the functional properties, give them new values, and thus expand the scope of applications. Figure 5 summarizes the utilization of protein–chitosan complexes in the food industry.

### 4.1. Microencapsulation

Microcapsules can be prepared using one or more materials that protect specific ingredients from external environmental factors, thereby enhancing the functionality. Compared to simple emulsion encapsulation, microcapsules offer higher encapsulation rates and antioxidant effects on the target ingredient [72]. To enhance the physicochemical properties of microcapsules, one approach is to perform protein–polysaccharide coacervation at a temperature that triggers the Maillard reaction. The impact of different temperatures (50 °C, 70 °C, and 90 °C) on the physicochemical properties of microcapsules was examined. It was found that higher coagulation temperatures can significantly enhance microencapsulation properties and reduce coagulation. When the condensation temperature rose successively from low (50 °C), medium (70 °C), to high (90 °C) temperature, the swelling rate of microcapsules decreased [72]. At elevated temperatures, the Maillard reaction occurs between soybean protein isolate and chitosan [73]. The generation of the Maillard reaction is beneficial to the stability of microcapsules and results in microcapsules with better flowability and higher packing density.

During the microencapsulation of probiotics, the wall material can protect the microorganisms. The wall material can consist of low-molecular-weight carbohydrates, proteins, or polysaccharides. Proteins can pass through stable membrane components to protect cells from damage. The combination of biopolymers (especially polysaccharides and proteins) with nanostructured materials is a novel and promising approach to maintaining the viability of probiotics in probiotic microcapsules and has been shown to be effective in maintaining the viability of the bifidobacterium during the digestive transition and in the intestinal tract [74]. Chitosan is considered to be a dietary fiber that protects microbes from gastric diseases and allows them to be released into the colon in appropriate amounts. Trypsin inhibitor (TTI) was encapsulated with chitosan whey protein nanoparticles. Chitosan mainly interacts with TTI to stabilize the system and binds to some anionic regions of whey protein isolate through fractional interaction. Compared to other formulations, the interaction between chitosan and whey protein improved the thermal stability of the microcapsules and showed excellent incorporation efficiency [75].

### 4.2. Composite Film and Coating

Due to its antioxidant, antibacterial, and water-insoluble properties, edible protein–chitosan films produced by the Maillard reaction have been investigated [76]. The use of composite films and coatings can effectively reduce the growth of undesirable microorganisms in fresh meat. Typically derived from proteins, polysaccharides, lipids or their mixtures, these films exhibit favorable properties such as transparency and mechanical strength when chitosan is incorporated due to its safety profile and excellent oxygen barrier properties. Chitosan has antimicrobial properties, but chitosan films are highly permeable to water vapor, leading to limitations in the application of chitosan films as an antimicrobial agent. It was found that the film formed by whey proteins and chitosan had good mechanical properties and barrier capacity, and the addition of organic acids significantly improved the antimicrobial capacity of the film [77]. In addition, applying this protective film to fresh turkey effectively prevented microbial spoilage while retarding the growth and development of pathogenic microorganisms [77]. In another study, the development of a composite film containing chitosan–sardine protein isolate for edible packaging improved the microbial stability of shrimp during refrigeration while reducing lipid peroxidation (*p* < 0.05) [78].

### 4.3. Antibacterial Aerogel

Aerogel is defined as an extremely light nanoporous material derived from a gel in which the liquid part has been replaced by gas [79]. As a carrier substrate, food-grade aerogels can protect loaded functional components from degradation, improve bioavailability, and provide well-controlled release. They can also be used as stabilizers, thickeners, and fillers in various food formulations. Currently, the combination of proteins and polysaccharides to develop aerogels has become a research hotspot, and molecular interactions can improve the mechanical properties, thermal insulation capacity, specific surface area, and density of aerogels [80,81]. The traditional absorbent pad is composed of polyethylene film and a nonwoven base, which has the characteristics of a low absorption rate and no antibacterial effect. A novel hyperabsorbent and antibacterial aerogel composed of isolated whey protein and chitosan was investigated. The aerogel can effectively prolong the shelf life of chicken to 7 days and can be used as a water-absorbing pad for meat preservation [13].

### 4.4. Food Grade Emulsion Conveying System

In the food industry, proteins are frequently utilized to stabilize emulsions and serve as carriers of nutrients and flavor. Protein–polysaccharide stabilized high internal phase Pickering emulsion (HIPE) has attracted extensive attention from researchers because of its excellent stability. It can be used as a delivery system to significantly improve the bioavailability of bioactive substances [82]. A large number of studies have reported that chitosan can be combined with soy protein isolate [83], cod protein [14], and phosphorylated perilla protein isolate [84] to develop HIPE and construct a food-grade emulsion delivery system. The properties of myofibrillar proteins (MPs) and chitosan (CS) complexes of Sparus macrocephalus were investigated. The results demonstrate that the MP/CS at a mixture ratio of 95:5 (*w*/*w*) has the potential to prepare HIPE. Furthermore, the MP/CS mixture can be employed to construct a food-grade emulsion delivery system with a high internal phase in the food industry [85]. The high intrinsic Pickering emulsion was stabilized by designing a cod protein–chitosan nanocomplex to deliver astaxanthin. The application of cod protein–chitosan nanocomplexes was demonstrated to form stable emulsions with a high internal phase, which significantly enhanced the chemical stability of astaxanthin [14].

## 5. Challenges and Solutions

### 5.1. Stability Problems of Protein–Chitosan Complexes

Although the protein–polysaccharide complex systems are relatively stable under certain conditions, they remain susceptible to external factors. The formation of protein–chitosan complexes relies mainly on non-covalent interactions such as electrostatic interactions and hydrogen bonds. As a result, the complexes are susceptible to a variety of factors, including pH, ionic strength, temperature, and protein–polysaccharide ratio. Temperature plays a crucial role in the coagulation process of the complexes. The formation of hydrogen bonds is favored at lower temperatures, while the exposure of hydrophobic groups is increased at higher temperatures due to alterations in protein structure. Although heat treatment can improve the emulsification properties of proteins to some extent, it can also lead to irreversible denaturation of proteins, decreasing their solubility and stability and thus aggregation [86,87]. High pressure may be an alternative processing method to heat treatment, and the use of it as a pretreatment may improve protein gel stability [88]. Proteins and chitosan are hotly researched as usable coatings for food protection, but the poor stability of chitosan limits its application. It was found that the introduction of cross-linking agents can effectively solve this problem [89]. Another finding also revealed that whey protein–carboxymethyl chitosan composite membranes in ratios of (whey protein/carboxymethyl chitosan = 75:25 and 50:50, *v*/*v*) treated with glutamine aminotransferase improved the water vapor barrier properties and mechanical properties [90]. In addition, proteins are usually hydrolyzed under acidic conditions, and ionic strength and pH affect the mixing of chitosan and proteins, thus affecting the stability of the complex. The study revealed that the soy protein–chitosan complex coagulates at a neutral pH. With the addition of salt ions (50, 100, and 200 mM), the electrostatic interaction between proteins and polysaccharides was weakened, creating an electrostatic shield and, thus, less stability. Protein–chitosan complexes show inferior performance compared to other materials. The main direction of future research will be to identify methods to maintain the stability of the complex in extreme environments [91].

### 5.2. Safety Problems of Protein–Chitosan Complexes

Chitosan and proteins can form edible films and coatings that inhibit the proliferation of harmful microorganisms through a Maillard reaction. Due to its potential application in food packaging, it has been extensively researched in the last decade [92]. Nevertheless, despite the potential benefits, protein–chitosan films may encounter certain challenges before entering the industrial market. Developing protein–chitosan complexes with the required properties and functions may result in reduced biosafety or biocompatibility or other potential side effects in the human body [93]. For instance, the cross-linking agents glutaraldehyde and epichlorohydrin are known to be toxic and are prevalent in most chitosan complex formulations, enhancing the functional properties of the complexes but raising concerns about potential toxicity. To resolve this limitation, the dialdehyde chitosan was prepared by a one-step reaction with sodium periodate, and the resulting chitosan membranes were less toxic than the sample crosslinked with glutaraldehyde, showing good solubility, mechanical properties, and thermal stability [94]. In addition, chitosan has low water solubility and is difficult to use directly, thus necessitating the production of chitosan derivatives. The solubility of chitosan with different DD degrees in a solvent is different, so chitosan with a certain DD value can be selected according to demand [95]. However, the use of potentially hazardous chemicals in the synthesis of these derivatives can pose an overall risk. In contrast to alkali and acid treatment, biological treatment and green synthesis can be employed as an alternative, with the objective of resolving the issue of environmental toxicity [93].

### 5.3. Problems of Compatibility and Persistence of Protein–Chitosan Complexes

The production of chitosan requires the use of a variety of acidic and basic reagents and elevated temperatures, which collectively result in a lengthy extraction process from crustacean shells. The sustainability of chitosan production is often a significant challenge. However, the implementation of effective strategies can effectively address these challenges. For example, the use of a deep eutectic solvent (a mild and environmentally friendly green solvent) to extract and process chitin shows the advantages of low toxicity, sustainability, biodegradability, and recyclability. However, due to its high cost, it cannot be used for mass production [94]. Moreover, chemical methods can be employed to modify the complexes in order to enhance the incompatibility between protein (e.g., whey protein isolate, quinoa protein) and chitosan. The production of chitosan can be extended by using microwave technology to reduce the extraction time of chitin [96]. Consequently, the practical implementation of protein–chitosan complexes is subject to certain challenges. However, according to the properties of different proteins, appropriate adjustment of the conditions of protein chitosan complex (such as pH value and ratio) can obtain ideal target products and achieve application [97,98].

## 6. Conclusions and Outlooks

This paper examines the interaction between protein and chitosan and its impact on functional properties, as well as the application of protein–chitosan complexes in the food industry. It also provides a comprehensive overview of the current challenges in the production of protein–chitosan complexes. The interaction between chitosan and protein is currently a subject of increasing interest. In future research, we can consider the following avenues: (1) The design of different complexes to meet the needs of the food industry by exploring the electrical properties of protein and chitosan. (2) Since the food system is not a single combination of polysaccharides and proteins, the formation and establishment of a ternary system (or even a multicomponent system) of chitosan with one protein, two proteins, or protein–chitosan complex will provide a more theoretical basis for food production. (3) Hydrolysates of protein–chitosan complexes have demonstrated a number of valuable functional properties, including high emulsification and specific binding of metal elements. Further study of complex hydrolysates will provide a broader platform for the comprehensive utilization of protein–chitosan.
